# Computational Methods in Drug Development: From Molecular Modeling to Artificial Intelligence Technologies

**DOI:** 10.3390/ph19081266

**Published:** 2026-08-11

**Authors:** Yunjie Zhao

**Affiliations:** Institute of Biophysics and Department of Physics, Central China Normal University, Wuhan 430079, China; yjzhaowh@ccnu.edu.cn

Advances in computational methods have significantly impacted protein design and drug development by offering innovative ways to accelerate the discovery and optimization of therapeutics [1,2]. Recently, molecular modeling and artificial intelligence have advanced in their ability to predict the structures of proteins, RNA, and drug molecules, as well as their complexes; identify new drug targets; and optimize drug–receptor interactions. Computational approaches are effective alternatives because experimental methods for determining molecular structures can be expensive and time-consuming. They also enhance our collective understanding of complex biological systems, enable high-throughput screening of large chemical libraries, and help to reduce time and costs compared to traditional methods. This Special Issue, “Computational Methods in Drug Development,” highlights these roles of computation in current pharmaceutical research and covers four key areas: understanding molecular mechanisms, drug discovery techniques, disease-focused therapeutic development, and future research directions.

Accurately understanding the structure and function of complex systems through experimental methods alone remains challenging [3]. Gao et al. explored techniques for predicting RNA–small-molecule binding sites [4]. Their review offers a timely summary of recent progress, systematically illustrating the history from physics-based strategies to integrated AI techniques. Traditional methods rely on limited data, whereas recent AI developments enable the integration of diverse features, leading to better predictions. The review explains the core principles behind different features, compares how features behave at binding versus non-binding sites, assesses method performance across different feature sets and test datasets, and highlights ongoing opportunities and challenges in flexibility prediction [5]. The p53 tumor suppressor protein contains intrinsically disordered N- and C-terminal domains. Ning et al. used microsecond-scale molecular dynamics simulations to investigate how the CTD and DNA binding regulate the allosteric mechanism of the p53 DNA-binding domain (DBD) [6]. Firstly, DNA binding induces conformational changes in the DBD, particularly in the S6-S7 loop and L1. Subsequently, the CTD directly interacts with DNA and influences the allosteric network, facilitating DBD binding to DNA. The allosteric mechanisms outlined here offer new insights into the functional roles of the p53 CTD and can guide the rational design of p53-targeting drugs. While a compound might show strong predicted binding, it can still fail due to unfavorable absorption, distribution, residence time, or clearance. Bueso-Bordils et al. [7] used pharmacokinetic equations to develop topological models to discover antibacterial compounds. This approach brings developability factors into the initial stages of computational screening, which is beneficial because the top-ranked compound based on target affinity is not always the best candidate for further development.

The process of developing inhibitors and prioritizing candidates involves complex computational methods. Jelčić et al. examined charged thienobenzo-1,2,3-triazoles as non-selective cholinesterase inhibitors, integrating chemical design, synthesis, anti-inflammatory testing, and computational analysis [8]. Their research emphasizes the promise of novel charged triazolinium salts as selective cholinesterase inhibitors with extra anti-inflammatory effects. De Souza et al. investigated the phytochemicals of Brazilian green propolis as potential B-Raf600E enzyme inhibitors using physicochemical analysis, ADME prediction, docking, molecular dynamics, and free-energy calculations [9]. Their work demonstrates how computational tools can help prioritize complex natural compounds for further testing. Buhlak et al. developed and evaluated 3,4-dihydroquinolin-2(1H)-one analogs as potential VEGFR2 inhibitors for glioblastoma therapy. Their approach included molecular docking and dynamics studies to analyze how these compounds interact with critical residues in the VEGFR2 binding pocket [10]. Overall, they show that AI and large-scale modeling are most effective when integrated into workflows that also consider chemical feasibility, target biology, and validation processes. AI improves candidate selection by aiding target identification, screening, optimization, repurposing, and clinical decision-making. Malheiro et al. reviewed AI’s potential in pharmaceutical innovation, emphasizing that it is not a simple solution but a tool that accelerates the search and prioritization processes [11]. AI has revolutionized drug discovery and development by enabling rapid, efficient analysis of extensive biological and chemical data, helping to identify new therapeutic compounds from initial research through clinical trials. However, there are still gaps in AI deployment, primarily due to a lack of regulation.

Scientists have focused on disease-centered therapeutic discovery, recognizing that disease biology is complex and seldom involves just one molecular interaction. Therapeutic outcomes are influenced by gene networks, pathway compensation, cellular states, and patient heterogeneity. Ghandikota and Jegga introduce a new computational framework that combines network analysis, statistical mediation, and deep learning to discover causal target genes and potential small-molecule drug candidates for repurposing [12]. Incorporating causal reasoning, their framework seeks to move beyond mere correlations toward testable hypotheses. Their study illustrates how molecular signatures associated with disease can guide target and drug prioritization. Likewise, in inflammatory disease models, Bissacotti et al. examined resveratrol’s anti-inflammatory effects via the P2X7/NLRP3 pathway, using in silico and in vitro evidence from activated microglia [13]. Docking analyses highlight potential molecular interactions, which cellular assays then test against biological responses. This iterative modeling and experimental process offers deeper insight than computational predictions alone, especially for compounds with multiple biological effects. Sharma et al. used a systems biology strategy that included protein–protein interaction (PPI) networks, molecular docking, and drug repurposing to identify host-targeted treatments for Oropouche virus [14]. These strategies are useful when time or resources limit direct antiviral development, but they must be carefully validated to prevent harmful disruptions to host pathways. Overall, computational drug development is viewed as a cohesive, integrated process of reasoning. Docking is most effective when combined with molecular dynamics, free-energy calculations, chemical synthesis, and experimental validation. Machine learning predictions improve when they utilize interpretable features, biological mechanisms, or validation data. Network pharmacology becomes more relevant when integrated with disease context and causal inference. Meanwhile, pharmacokinetic and developability models help to avoid early prioritization based solely on target affinity. Furthermore, RNA-binding site prediction and allosteric simulations expand the types of systems suitable for computational analysis [15,16].

Although progress has been achieved, benchmark quality for AI and machine learning models trained on retrospective data remains a concern. Challenges such as data leakage, biased negative samples, a narrow chemical scope, and inconsistent experimental annotations can result in overly optimistic performance evaluations. Interpretability remains challenging. Models that prioritize compounds, targets, or pathways without clarifying their reasoning can be helpful for preliminary screening but are less effective for fine-tuning based on mechanisms. Biological systems function across various levels, with molecular binding being just one aspect of the therapeutic response. Outcomes are also affected by factors like tissue exposure, metabolism, immune environment, compensatory signaling, and patient variability. As a result, validation should be incorporated into the computational workflow rather than viewed as an endpoint. Future advancements are expected to include physics-informed machine learning models that integrate molecular graphs, sequences, 3D structures, omics data, and pharmacology [17]. This Special Issue emphasizes that computation complements experiments rather than replacing them, supporting the systematic discovery process. By connecting molecular mechanisms, inhibitor design, disease contexts, and validation, computational methods help researchers to develop more accurate hypotheses and make more informed decisions.
